# Epoxyeicosatrienoic Acid Analog and 20-HETE Antagonist Combination Prevent Hypertension Development in Spontaneously Hypertensive Rats

**DOI:** 10.3389/fphar.2021.798642

**Published:** 2022-01-17

**Authors:** Iwona Baranowska, Olga Gawrys, Agnieszka Walkowska, Krzysztof H. Olszynski, Luděk Červenka, John R. Falck, Adeniyi M. Adebesin, John D. Imig, Elżbieta Kompanowska-Jezierska

**Affiliations:** ^1^ Department of Renal and Body Fluid Physiology, Mossakowski Medical Research Institute, Polish Academy of Science, Warsaw, Poland; ^2^ Department of Immunology, Medical University of Warsaw, Warsaw, Poland; ^3^ Center for Experimental Medicine, Institute for Clinical and Experimental Medicine, Prague, Czechia; ^4^ Behavior and Metabolism Research Laboratory, Mossakowski Medical Research Institute, Polish Academy of Sciences, Warsaw, Poland; ^5^ Department of Biochemistry, University of Texas Southwestern Medical Center, Dallas, TX, United States; ^6^ Department of Pharmacology and Toxicology, Medical College of Wisconsin, Milwaukee, WI, United States.

**Keywords:** epoxyeicosatrienoic acids, primary hypertension, EET analog, 20-HETE antagonist, spontaneously hypertensive rats

## Abstract

Numerous studies indicate a significant role for cytochrome P-450-dependent arachidonic acid metabolites in blood pressure regulation, vascular tone, and control of renal function. Epoxyeicosatrienoic acids (EETs) exhibit a spectrum of beneficial effects, such as vasodilatory activity and anti-inflammatory, anti-fibrotic, and anti-apoptotic properties. 20-Hydroxyeicosatetraenoic acid (20-HETE) is a potent vasoconstrictor that inhibits sodium reabsorption in the kidney. In the present study, the efficiency of EET-A (a stable analog of 14,15-EET) alone and combined with AAA, a novel receptor antagonist of 20-HETE, was tested in spontaneously hypertensive rats (SHR). Adult SHR (16 weeks old) were treated with two doses of EET-A (10 or 40 mg/kg/day). In the following experiments, we also tested selected substances in the prevention of hypertension development in young SHR (6 weeks old). Young rats were treated with EET-A or the combination of EET-A and AAA (both at 10 mg/kg/day). The substances were administered in drinking water for 4 weeks. Blood pressure was measured by telemetry. Once-a-week observation in metabolic cages was performed; urine, blood, and tissue samples were collected for further analysis. The combined treatment with AAA + EET-A exhibited antihypertensive efficiency in young SHR, which remained normotensive until the end of the observation in comparison to a control group (systolic blood pressure, 134 ± 2 *versus* 156 ± 5 mmHg, respectively; *p* < 0.05). Moreover the combined treatment also increased the nitric oxide metabolite excretion. Considering the beneficial impact of the combined treatment with EET-A and AAA in young rats and our previous positive results in adult SHR, we suggest that it is a promising therapeutic strategy not only for the treatment but also for the prevention of hypertension.

## Introduction

According to WHO, hypertension is one of the most significant risk factor for all-cause morbidity and mortality worldwide, and it is responsible for approximately 10 million deaths globally every year (World Health Organization, 2013; Stanaway et al., 2018). This multifactorial disease is a result of the complex interplay between environmental and pathophysiological factors involving multiple systems as well as genetic predispositions (Oparil et al., 2018). Elevated sympathetic activity, inflammation, and, most of all, over-activity of the renin–angiotensin system are the main factors contributing to the development of hypertension (Imig, 2019). Except for the often-overlooked encouragement for healthy lifestyle choices, a few successful pharmacological antihypertensive therapies have been introduced, including diuretics, beta-blockers, ACE inhibitors, angiotensin II (ANG II) receptor blockers, and calcium channel blockers (Unger et al., 2020). However, since morbidity and mortality related to hypertension increase continuously each year, a deeper understanding of the underlying mechanisms responsible for blood pressure elevation and novel antihypertensive therapies are still in great demand.

The role of cytochrome P-450 (CYP-450)-dependent metabolites of arachidonic acid (AA) in blood pressure regulation has been explored for more than a decade (Imig, 2010; Imig, 2015; Imig, 2019). CYP-450 epoxygenases (mainly CYP2C and CYP2J) produce epoxyeicosatrienoic acids (EETs), which can be metabolized to less biologically active diols by soluble epoxide hydrolase (sEH) enzymes. EETs were recently proved to possess beneficial properties on renal and cardiovascular systems as demonstrated in several animal models of diseases, including our own studies (Elmarakby, 2012; Neckar et al., 2012; Capdevila and Wang, 2013; Capdevila et al., 2014). An excellent review summarizing the role of EETs in blood pressure regulation (Imig, 2019) was published recently, which clearly points that EET deficiency can lead to hypertension and renal dysfunction, thus giving a rationale to EET supplementation as a novel therapeutic option.

Water-soluble, orally active, and metabolically resistant EETs analogs were developed and tested (Falck et al., 2003; Imig et al., 2010; Khan et al., 2013; Alánová et al., 2015; Campbell et al., 2017), including the analog of the most abundant 14,15-EET isoform (called EET-A). This compound possesses antihypertensive and antiarrhythmic properties in Ren-2 transgenic rats (TGR), inhibits sodium transport, protects vascular endothelial function, and blocks renal tubular sodium channel in ANG II-dependent hypertension (Khan et al., 2014, Hye Khan et al., 2014; Jíchová Š. et al., 2016; Červenka et al., 2018).

However, most of the above-mentioned studies were performed on rather single-factorial animal models of hypertension, such as TGR rats, which are strictly ANG II dependent. It is generally accepted that essential hypertension is a complex, polygenic disease (Neutel and Smith, 1999). Thus, in our own recent study (Gawrys et al., 2020a), we decided to evaluate the effectiveness of EET-A in spontaneously hypertensive rats (SHR), which better mimic human primary hypertension (Doris, 2017; Mohammed-Ali et al., 2017). Surprisingly, we found that EET-A given alone to adult hypertensive SHR (10 mg/kg/day) did not exhibit any antihypertensive properties. Hence, in the present study, we decided to test a four-times-higher dosage of EET-A (40 mg/kg/day) to establish if the previously used dosage was simply too low to counteract all the detrimental factors present in SHR (series 1).

A second goal of the present study relates to the most important finding of our previous study in adult SHR (Gawrys et al., 2020a)—that the treatment with EET-A and 20-hydroxyeicosatetraenoic acid (20-HETE) receptor antagonist (AAA) demonstrated to be a very powerful antihypertensive combination. 20-HETE is a very unique AA metabolite with both pro- and antihypertensive activities (Nowicki et al., 1997; Ward et al., 2004; Wu et al., 2014). Interestingly, both the stimulation of its production (Jíchová Š et al., 2016) and the inhibition of its action (Sedláková et al., 2018) can cause beneficial effects. Moreover, we have recently shown that, after acute infusion of both compounds, a significant improvement in renal hemodynamics is observed, with a subsequent reduction in blood pressure (Walkowska et al., 2021). Because of our very promising results with EET-A and AAA, we decided to test its potential to prevent the development of hypertension in young rats in pre-hypertensive stage (series 2). Since essential hypertension is partially related to autonomous nervous system imbalance, especially in the early stages (Carthy, 2014), an additional analysis of heart rate variability (HRV) was performed.

The treatment was applied in drinking water for four consecutive weeks. Adult SHR received a higher dose of EET-A (40 mg/kg/day), and young SHR received EET-A alone or EET-A combined with AAA (10 mg/kg/day each). Blood pressure was measured by telemetry, and observations in metabolic cages (combined with urine and blood sampling) were performed once a week.

## Materials and Methods

### Chemicals and Reagents

EET-A [disodium (Z)-(13-(3-pentylureido) tridec-8-enoyl)-l-aspartate], a 14,15-EET analog, was given in drinking water, and its concentration was adjusted in such a way that the daily dose was either 10 mg/kg/day (EET-A) or a high dose of 40 mg/day/kg (EET-A HD). 20-HETE receptor antagonist {disodium [(6Z,15Z)-20-hydroxyeicosa-6,15-dienoyl]-l-aspartate; AAA} was also given in drinking water at a concentration adjusted to yield a daily dose of 10 mg/kg/day, based on our recent studies (Gangadhariah et al., 2014; Sedláková et al., 2018; Gawrys et al., 2020a). The EET-A and AAA were designed and synthesized in the laboratory of JRF.

### Experimental Animals

The experimental procedures were approved by the I Ethical Committee for Animal Experimentation (Warsaw), which follows the European Directive 2010/63/EU on the protection of animals used for scientific purposes. Male adult (16 weeks old; mean body weight, 313 ± 5 g; *n* = 18) and young (6 weeks old; mean body weight, 123 ± 4 g; *n* = 21) SHR, bred at the Animal House of Mossakowski Medical Research Institute, Polish Academy of Sciences, were fed *ad libitum* a standard diet (0.25% Na w/w, SSNIFF GmbH, Soest, Germany) and had free access to drinking water during the whole experiment. The animals were housed at two per cage in a conventional animal room with controlled temperature (24 ± 2°C) and a 12/12 h light–dark cycle. During the first few weeks, the rats were allowed to get accustomed to the new housing, operating personnel, and procedures.

The rats were implanted with telemetry transmitters (TA11PA-C10 dedicated for young animals or TA11PA-C40 for adults; Data Sciences International, St. Paul, United States) under aseptic conditions and under isoflurane anesthesia (IsoVet^®^, Piramal Healthcare, United Kingdom) at 4% in the induction phase and maintained by mask inhalation at 2–1.5% during the procedure (Combi-vet^®^ system, Rothacher Medical GmbH, Heitenried, Switzerland). The cannula of the transmitter was implanted into the aorta, and the body of the transmitter was placed inside the peritoneal cavity and fixed to the abdominal muscle wall. Metacam (0.4 mg/kg BW, Boehringer, Ingelheim, Germany) and Baytril (10 mg/kg/day, Bayer, Leverkusen, Germany) were used as post-operative analgesia and to prevent infection, respectively. All the procedures were previously described in detail by us (Gawrys et al., 2018; Gawrys et al., 2020a; Gawrys et al., 2020b).

The basal blood pressure (BP) was measured continuously for a few days, and then the treatment was applied for four consecutive weeks. To eliminate the impact of circadian rhythm, telemetry data were appropriately acquired from each group at the same time intervals. The average values were calculated from 24-h continuous recordings, from 6 to 6 a.m. at 12/12-h light/dark cycle on the days without any other procedures (metabolic cages or blood sampling); therefore, the blood pressure measurements are presented every 3 or 4 days. Moreover, 24-h observations in metabolic cages were performed once a week on days 0, 7, 14, 21, and 28. Urine samples and blood samples were collected once a week. At the end of the experiments, the animals were euthanized, and tissues were collected for further analysis.

### Experimental Design

#### Series 1: Adult rats in an established phase of hypertension (16 weeks old) and receiving only EET-A in two doses:

1. EET-A (10 mg/kg/day, *n* = 6)
2. EET-A HD (40 mg/kg/day, *n* = 6)
3. Control group (drinking water, *n* = 6)

#### Series 2: Young rats (6 weeks old) in the development stage of the disease and receiving EET-A alone or combined with AAA:

1. EET-A (10 mg/kg/day, *n* = 7)
2. EET-A + AAA (both at 10 mg/kg/day, *n* = 6)
3. Control group (drinking water, *n* = 7)

### Heart Rate Variability Analysis

HRV analysis was performed using Ponemah 6.32 software (Data Science International, St. Paul, Minnesota, United States). An approach proposed by Thireau *et al*. (2008; Battault et al., 2018), suitable for rodents, was applied. The HRV parameters were calculated for days 0 and 28 during fulltime recordings (*i*.*e*., over light and dark periods). The telemetry recordings were validated to avoid false positive detections and missed beats, and the normal beat-to-beat intervals (NN intervals) were determined.

The time-domain parameters were calculated from the entire analysis period and included the following: (1) mean NN intervals (in ms), (2) standard deviation of all NN intervals (SDNN, in ms), (3) root mean square of successive differences between normal heartbeats (in ms), and (4) percentage of normal consecutive NN intervals differing by >*x* ms (pNNx, %). For humans, typically pNN50 is determined; since the heart rate of a rat is much higher than that of a human, there is a need to apply a lower number. However, there are no standard pNNx values for rats. In this research, pNN5 was calculated as proposed by Aubert *et al*. (1999).

The frequency-domain parameters were calculated from short intervals. We selected and analyzed one 3-min period with no erratic fluctuations for every 30 min. These sections were filtered using a Hanning window. Predefined spectral bands adjusted to the rat were used as follows: (1) the very low frequency band (VLF) under 0.2 Hz, (2) the low frequency band (LF) ranging between 0.2 and 0.74 Hz, and (3) the high frequency band (HF) ranging between 0.74 and 2.50 Hz (Aubert et al., 1999; Vega-Martínez et al., 2014). The power spectrum density was expressed in absolute values (ms^2^ for pulse interval, for VLF) and in normalized units (nu, for normalized LF and normalized HF), which represents the relative value of each power component in proportion to the total power minus the very low frequency component (Task Force of the European Society of Cardiology and the North American Society of Pacing and Electrophysiology, 1996). Additionally, the LF–HF ratio and total spectral power (TP) were also calculated.

### Analytical Procedures

The freezing point depression method (Osmomat^®^ 030 M, Gonotec, Berlin, Germany) was used to measure plasma and urine osmolality. Sodium concentrations were determined by flame photometry (PFP7/C, Jenway Ltd., Stone, United Kingdom). Commercially available kits were used to assess the levels of VEGF-A (cat: K5365, BioVision, San Francisco, United States), albumin (Rat Albumin ELISA Kit, cat: E-25AL, Portland, United States), nitric oxide metabolites (Nitrate/Nitrite Colorimetric Assay Kit, cat: 780,001, Cayman Chemical, Michigan, United States), creatinine (Creatinine Assay Kit, cat: KA0849, Abnova, Taipei City, Taiwan), and interleukin 10 (IL-10; Rat ELISA Kit, cat: BMS629, Thermo Fisher Scientific).

In selected groups (of the most interest), the renal concentration of 23 cytokines was measured with Bio-Plex Pro Rat Cytokine 23-Plex Immunoassay (cat. no. 12005641, Bio-Rad Laboratories Inc., California, United States). The tissue samples were homogenized on ice in glass homogenizers with TritonX-100 (final concentration 1%, cat. no T8787, Sigma-Aldrich) in phosphate-buffered saline containing a mixture of protease inhibitors (cat. no P8340, Sigma Aldrich). The ratio of tissue weight to buffer was 1:10. After homogenization, the suspension was centrifuged (20,000 *g*, 10 min, 4°C), and the supernatants were collected and mixed with Sample Diluent (at a ratio of 1:11.5). Thereafter, the procedure provided by the manufacturer was followed. All measurements were performed in duplicates. The concentration of each cytokine was expressed per gram of protein (the total protein concentration was measured by Pierce™ BCA Protein Assay Kit, cat. no. 23225, Thermo Scientific™).

### Statistical Analysis

All values are expressed as means ± SEM. Graph-Pad Prism software (Graph Pad Software, San Diego, California, United States) was used for statistical analysis of the data. Multiple-group comparisons were performed by multiple *t*-test and one- or two-way analysis of variance, followed by Tukey’s *post-hoc* test (between groups) or Bonferroni’s multiple-comparisons test (within each group) as appropriate. Values exceeding the 95% probability limits (*p* < 0.05) were considered statistically significant.

## Results

### Series 1: Adult Rats in an Established Phase of Hypertension (16 Weeks Old) and Receiving Only EET-A in Two Doses or Solvent

**FIGURE 1:**
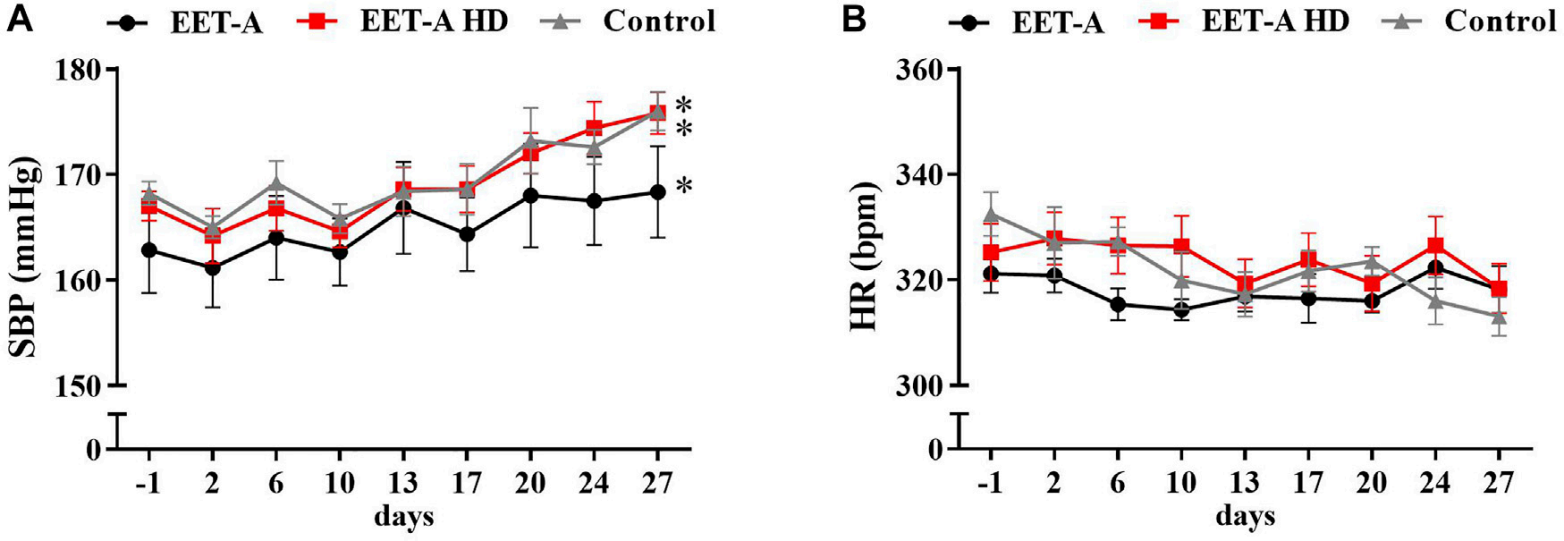
Time course of **(A)** systolic blood pressure and **(B)** heart rates in adult spontaneously hypertensive rats receiving epoxyeicosatrienoic acid analog in two doses at 10 mg/kg/day (*n* = 6, EET-A) and 40 mg/kg/day (EET-A HD, *n* = 6); the control group received water (*n* = 6). Values are expressed as means ± SEM. *p <0.05 *vs*. baseline values (day 1) within each group by two-way analysis of variance, followed by Bonferroni’s multiple-comparisons test (not significant between groups).

**FIGURE 2:**
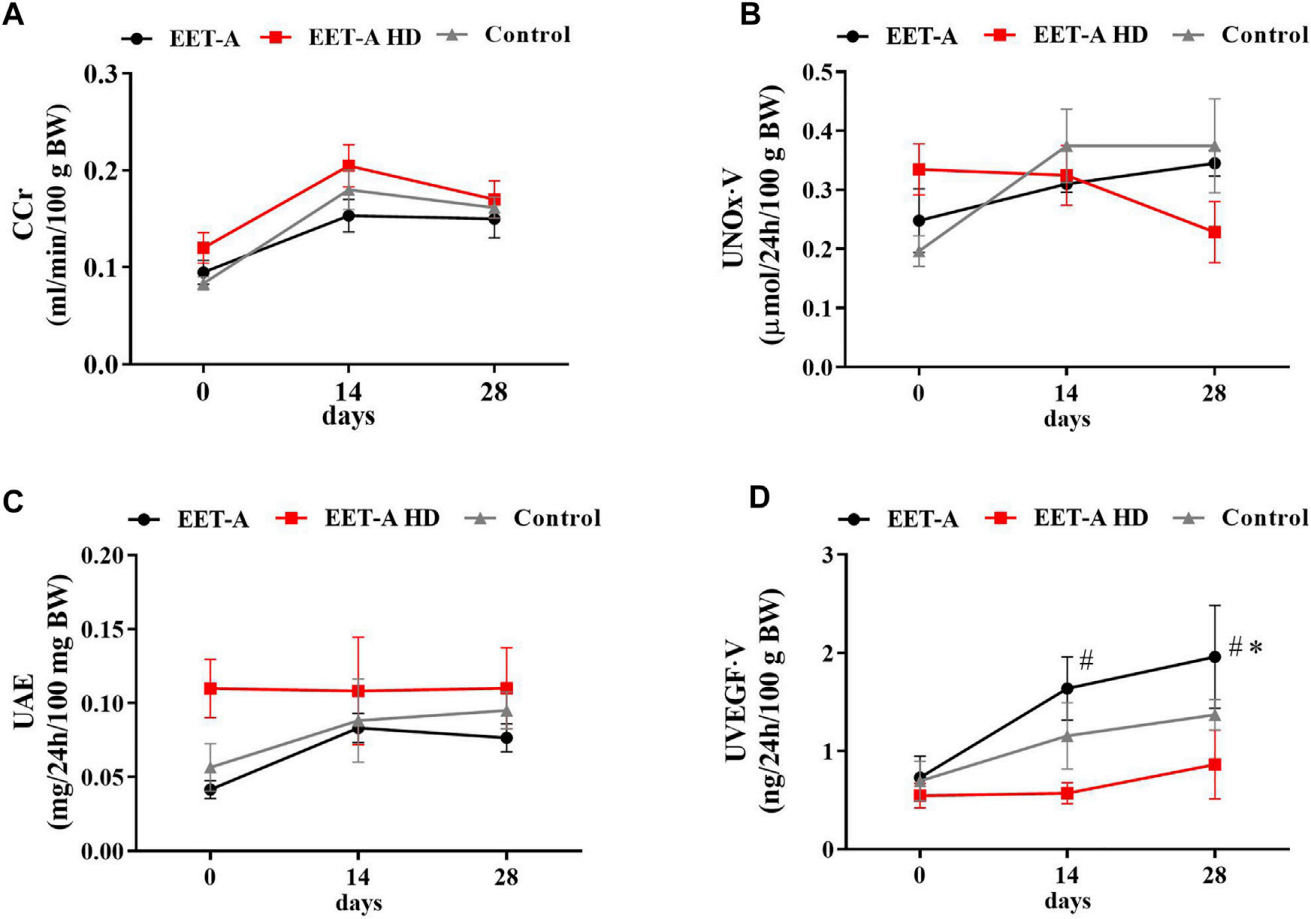
**(A)** Creatinine clearance, **(** **B** **)** nitric oxide metabolites excretion, **(C)** urinary excretion of albumin, and **(D)** urinary excretion of vascular endothelial growth factor type A measured on days 0, 14, and 28 in adult spontaneously hypertensive rats receiving epoxyeicosatrienoic acid analog in two doses at 10 mg/kg/day (*n* = 6, EET-A) and 40 mg/kg/day (EET-A HD, *n* = 6); the control group received water (*n* = 6). Values are expressed as means ± SEM. *p <0.05 *vs*. baseline values (day 0) within the EET-A group by two-way analysis of variance, followed by Bonferroni’s multiple-comparisons test; #*p* <0.05 EET-A *vs*. EET-A HD at the same time point by two-way analysis of variance, followed by Tukey’s *post-hoc* test.

None of the applied doses of EET-A resulted in systolic blood pressure (SBP) reduction (Figure 1A). Additionally, the heart rates were not influenced by the treatment (Figure 1B). Data on mean blood pressure (MBP) and diastolic blood pressure (DBP) are available in the supplementary files (Supplementary Figure S1). There were no clear differences in HRV (both time and frequency domains) in adult rats—neither between groups nor between days 28 and 0 (Supplementary Tables S1, S2). The majority of the measured parameters did not differ between groups, and the EET-A analog in both applied doses did not cause any changes in metabolic, plasma, or excretory parameters (Supplementary Tables S3, S4). The renal and cardiovascular biomarkers were also measured and standardized on 100 mg of body weight (Figure 2). The left ventricular hypertrophy and weight of the kidneys were also not affected by any of the treatments (Supplementary Table S5).

Creatinine clearance (CCr), urinary excretion of albumin (UAE), and nitric oxide metabolite excretion did not differ between groups (Figures 2A–C). The urinary excretion of vascular endothelial growth factor type A (U_VEGF_·V) increased significantly, but only after treatment with the standard dose of EET-A (10 mg/kg/day; Figure 2D). The plasma level of interleukin 10 (P_IL-10_) was similar in all groups and was not influenced by treatment with both doses of the analog (Table 1). The level of IL-10 in the kidney, similarly to the plasma level, did not differ between the groups. The renal concentration of interleukin 2, interleukin 4, interleukin 17a, macrophage colony-stimulating factor, monocyte chemoattractant protein 3, interferon gamma, and granulocyte colony-stimulating factor significantly increased after treatment with the high dose of EET-A (Supplementary Table S6).

**TABLE 1:** Plasma level of interleukin 10 (IL-10) on days 0, 14, and 28 in spontaneously hypertensive rats (SHR): adult SHR receiving oral treatment with epoxyeicosatrienoic acid analog at a dose of 10 mg/kg/day (EET-A) or 40 mg/kg/day (EET-A HD) and young SHR treated with epoxyeicosatrienoic acid analog (EET-A) alone or combined with AAA, a 20-hydroxyeicosatetraenoic receptor antagonist (EET-A + AAA), both at a dose of 10 mg/kg/day.

**IL-10 (pg/ml)**		**Day 0**	**Day 14**	**Day 28**
Adult SHR	EET-A	70 ± 12	56 ± 14	54 ± 12
	EET-A HD	58 ± 10	53 ± 14	54 ± 10
	Control	72 ± 13	56 ± 15	74 ± 19
Young SHR	EET-A	68 ± 16	42 ± 6	34 ± 5
	EET-A + AAA	57 ± 15	48 ± 9	39 ± 9
	Control	44 ± 10	56 ± 7	72 ± 13

Values are expressed as means ± SEM (*n* = 6 in each group); not significant.

### Series 2: Young Rats (6 Weeks Old) in the Development Stage of the Disease and Receiving EET-A Alone or Combined With AAA

**FIGURE 3:**
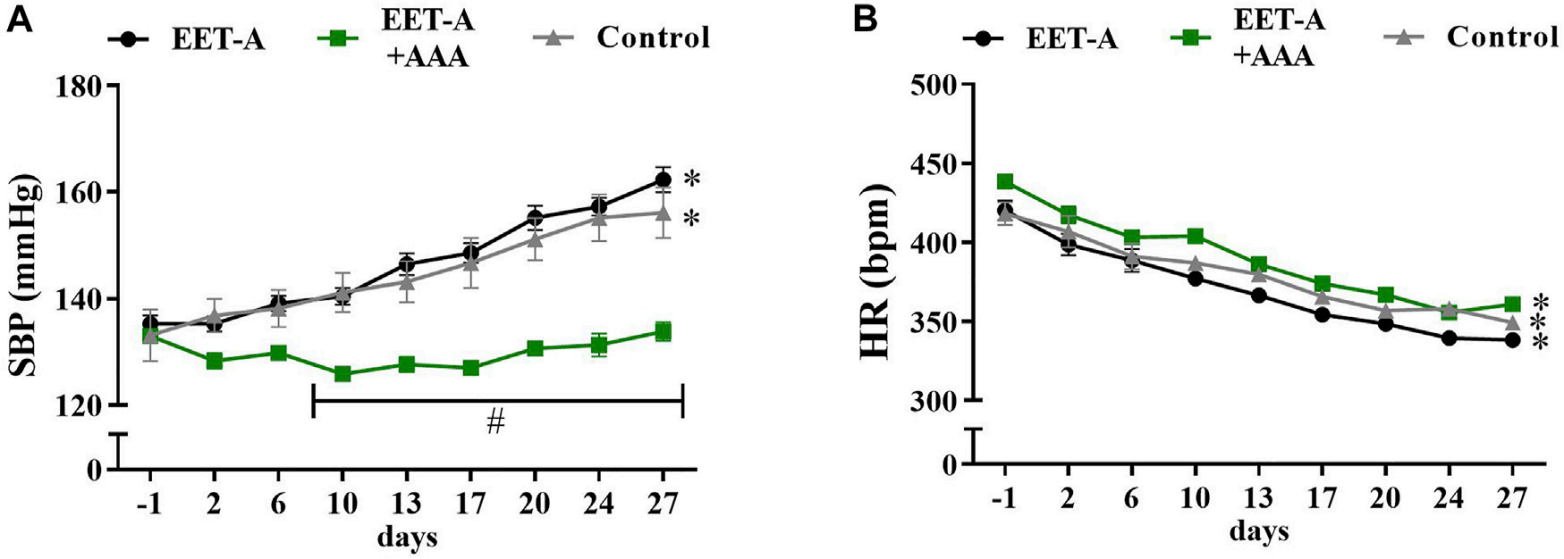
Time course of **(A)** systolic blood pressure and **(B)** heart rates in young spontaneously hypertensive rats receiving epoxyeicosatrienoic acid analog alone (EET-A, *n* = 7) or combined with 20-HETE receptor antagonist (EET-A + AAA, *n* = 6), both at a dose of 10 mg/kg/day; the control group received water (*n* = 7). Values are expressed as means ± SEM. *p <0.05 *vs*. baseline values (day 1) within each group by two-way analysis of variance, followed by Bonferroni’s multiple-comparisons test; #*p* <0.05 EET-A + AAA *vs*. EET-A and the control group at the same time point by two-way analysis of variance, followed by Tukey’s *post-hoc* test.

The combination of EET-A and AAA attenuated the development of hypertension in young rats (Figure 3A), *i*.*e*., the SBP values in this group were significantly lower than in the other groups (*p <0.05 starting from day 10 until the end of the observation; the graphs for MBP and DBP are available in Supplementary Figure S1). Additionally, there were no significant differences within the EET-A + AAA group (the rats remained normotensive: SBP before the treatment 133 ± 1 *vs*. in the end of the experiment 134 ± 2 mmHg, NS), whereas in the control and EET-A-treated groups, the SBP increased progressively to a clearly hypertensive level, similar to the blood pressure of adult rats in the established phase of the disease used in series 1 (a comparison of the blood pressure in young and adult rats is available in Supplementary Figure S1). In all three groups, the heart rates decreased during the 4-week observation, which is a normal physiological phenomenon in this rat strain. In all groups of young rats, independently of the treatment, the values of NN interval, SDNN, VLF, and TP were higher on day 28 than on day 0 (Supplementary Tables S7, S8). We observed some differences in HRV only after the combined treatment with EET-A + AAA. Firstly, the NN interval for EET-A + AAA was significantly lower than for the EET-A group (165.3 ± 1.4 *vs*. 176.7 ± 2.4, respectively; *p* = 0.04; Supplementary Table S7); however, the comparison of the increments (*i*.*e*., values from day 28 minus values from day 0) was insignificant between both groups. Secondly, the VLF for EET-A + AAA was lower than in the other groups; however, it was significant only *versus* the control group (EET-A: 25.3 ± 1.4; EET-A + AAA: 20.3 ± 2.0*; control: 26.8 ± 1.6; *p <0.05 EET-A + AAA *vs*. control; EET-A + AAA *vs*. EET-A, *p* = 0.0735; Supplementary Table S8). NN and SDNN were increased on day 28 compared to day 0 of the experiment in all groups, which could be an effect of the maturation of the animals, followed by the development of hypertension.

Similar to adult rats, none of the treatments significantly changed the metabolic, plasma, and excretory parameters (Supplementary Tables S9, S10). In many cases, the parameters such as diuresis, water intake, food intake, and feces excretion increased significantly as a result of normal development and maturation of young rats. More importantly, there were no relevant differences between the experimental groups. Data on the weight of organs collected at the end of the 4-week observation period also did not differ between the treated and control groups (Supplementary Table S11).

**FIGURE 4:**
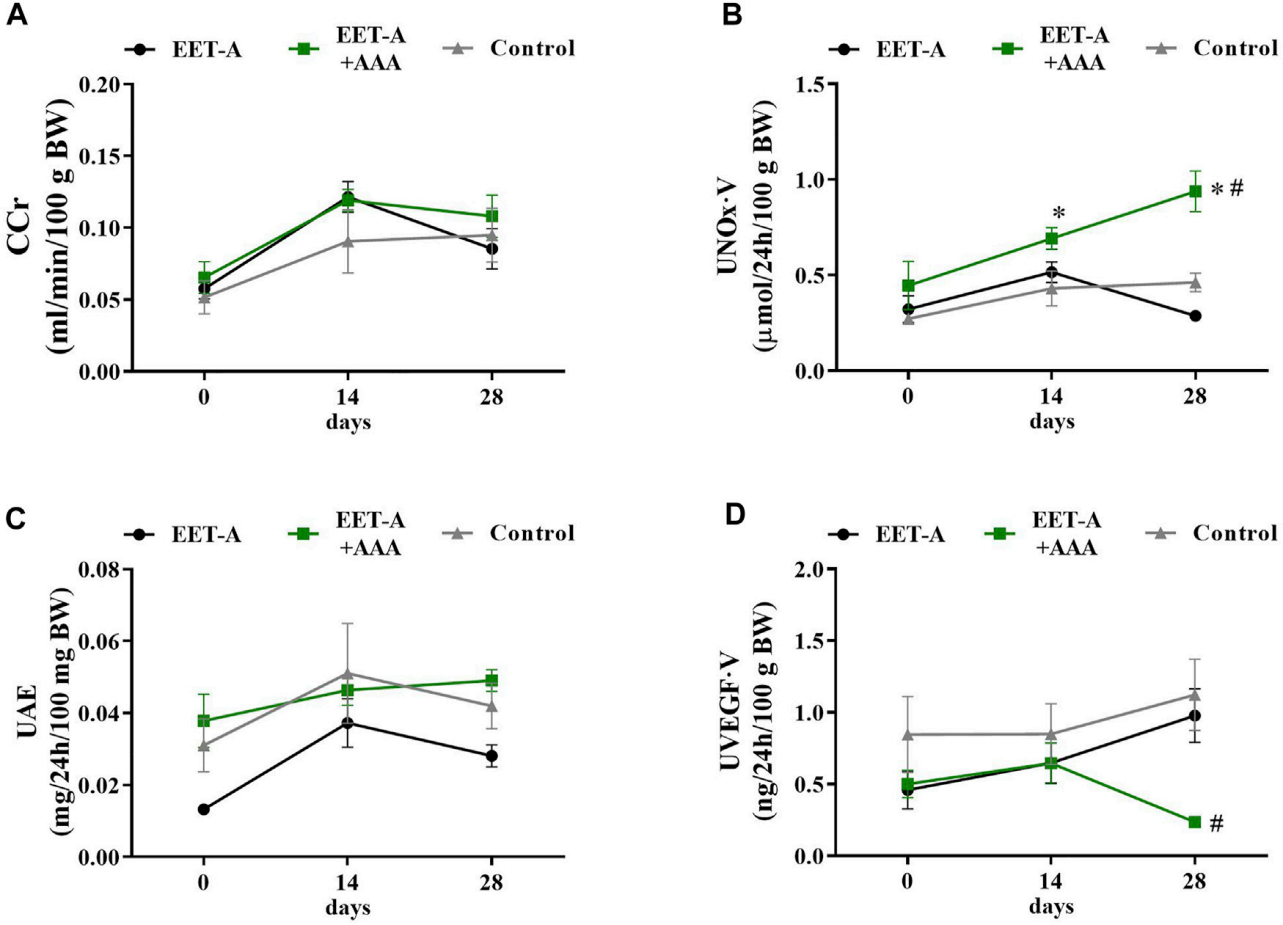
**(A)** Creatinine clearance, **(B)** nitric oxide metabolite excretion, **(C)** urinary excretion of albumin, and **(D)** urinary excretion of vascular endothelial growth factor type A measured on days 0, 14, and 28 in young spontaneously hypertensive rats receiving epoxyeicosatrienoic acid analog alone (EET-A, *n* = 7) or combined with 20-HETE receptor antagonist (EET-A + AAA, *n* = 6), both at a dose of 10 mg/kg/day; the control group received water (*n* = 7). *p <0.05 *vs*. baseline values (day 0) within each group by two-way analysis of variance, followed by Bonferroni’s multiple-comparisons test; #*p* <0.05 EET-A + AAA *vs*. EET-A and the control group at the same time point by two-way analysis of variance, followed by Tukey’s *post-hoc* test.

All the assessed renal biomarkers were still in the range for healthy, normotensive rats. CCr and UAE were on the same level in all groups (Figure 4 A and C, respectively), and none of the treatments influenced these renal biomarkers. It is noteworthy that CCr and UAE were lower in young rats compared to adult animals. Importantly, treatment with the combination of EET-A + AAA significantly increased the nitric oxide metabolite excretion in young SHR. The observed increase was significant after 14 and 28 days (*versus* the basal values on day 0 within the EET-A + AAA group) and substantially different in comparison with the EET-A alone and control groups at the end of the experiment (Figure 4B). In the same group treated with the combination of EET-A and AAA, the urinary excretion of VEGF-A was significantly lower in comparison to the EET-A alone and control groups at the end of the experiment (Figure 4D). The plasma level of IL-10 increased slightly in the control group, and despite the lack of statistical differences, the decreasing trend for both groups receiving the treatment was different than in the control group (Table 1). Treatment with EET-A + AAA did not affect the concentration of any of the measured cytokines in kidney tissue (Supplementary Table S10).

## Discussion

The main important finding of this study is that the combined treatment with 14,15-EET analog (EET-A) and AAA prevented the blood pressure increase in young spontaneously hypertensive rats.

### Series 1: Adult Rats in an Established Phase of Hypertension (16 Weeks Old) and Receiving Only EET-A in Two Doses

A detailed analysis of the results obtained in adult rats confirmed our previous finding (Gawrys et al., 2020a) that EET-A alone is not effective in SHR even in a substantially higher dosage (40 mg/day/kg). We did not observe any differences in the measured parameters between all groups (with both dosages and the control group). The analysis of HRV did not reveal any meaningful changes between groups and will be discussed below in relation to young rats.

To test the effectiveness of the EET-A analog on inflammatory processes, we decided to analyze the palette of interleukins and growth factors only in selected groups with the most promising treatment regimen. The elevated levels of some cytokines in the EET-A HD group may suggest some negative effect of the high dose of the analog; it is likely that, instead of reducing the inflammatory process characteristic of SHR, it has been slightly increased, but the exact mechanisms are difficult to define. We hypothesize that the slightly elevated inflammation state and the lack of antihypertensive effectiveness of EET-A might be related to the central activity of the analog in the brain, where it can penetrate more easily due to a disrupted blood–brain barrier (BBB), which is already a well-known phenomenon in young SHR (Ueno et al., 2004). It has been proven that the peripheral effectiveness of EETs is paradoxically distinct from their activity in the brain (Sellers et al., 2005). It was shown that the intracerebroventricular inhibition of sEH, which changes the balance between EETs and less potent DHETs in favor of EETs, led to a significant increase in BP in SHR (Sellers et al., 2005). This elevation of blood pressure was probably caused by the depression of the baroreceptor reflexes and also led to an increase in reactive oxygen species production. The SHR strain is considered the best model to mimic primary human hypertension considering a lot of shared features, such as similar dynamics of the disease and the activity of crucial systems, such as the sympathetic nervous system (SNS) and the renin–angiotensin–aldosterone system (RAAS). Therefore, it is the most commonly used strain to study new antihypertensive drugs (Sellers et al., 2005; Lin et al., 2016; Mohammed-Ali et al., 2017). However, if our hypothesis is correct, *i*.*e*., the peripheral activity of EET-A is counterbalanced by its central action, it might explain the lack of antihypertensive effects in SHR, which was previously observed in other experimental models, such as TGR or angiotensin II-dependent hypertension (Khan et al., 2014; Hye Khan et al., 2014; Jíchová Š. et al., 2016; Červenka et al., 2018).

Another interesting but somehow confusing finding is with regard the excretion rate of vascular endothelial growth factor A (VEGF-A). VEGF was first discovered as a tumor-derived soluble factor responsible for angiogenesis and endothelial cell permeability (Apte et al., 2019). Very quickly after introducing VEGF-targeted anti-cancer therapies, hypertension and proteinuria occurred as dominant adverse effects (Wasserstrum et al., 2015; Apte et al., 2019). The excretion rate of VEGF-A increased, but only after treatment with the “standard dose” of EET-A (10 mg/day/kg). After careful consideration (together with the analysis of the results obtained in young rats; see below for more details), we are not able to provide any firm explanation to this phenomena, but it seems that this marker is quite susceptible to a variety of factors and not as reliable as we thought. Therefore, we dismiss our previous hypothesis regarding its role in CYP450-dependent metabolites of arachidonic acid pathways of blood pressure regulation.

Since we did not observe any beneficial effects of EET-A administration (even with a much higher dose) and considering the very promising results of our previous studies on both AA metabolites, *i*.*e*., EET-A and AAA in adult rats (Gawrys et al., 2020a), we decided to test if the combined treatment will be effective in preventing the blood pressure increase in young spontaneously hypertensive rats.

### Series 2: Young Rats (6 Weeks Old) in the Development Stage of the Disease and Receiving EET-A Alone or Combined With AAA

The combined treatment with EET-A and AAA substantially prevented the development of hypertension in young spontaneously hypertensive rats. The SBP values in this group were significantly lower than in the other groups, and there were no significant differences within the EET-A + AAA group (the rats remained normotensive). In the control group and the group treated with EET-A only, the SBP increased progressively to a clearly hypertensive level, similar to the blood pressure of adult rats in an established phase of the disease. We did not observe any differences in HR changes between the treatment and control groups. We would expect that, parallel to the BP differences, *i*.*e*., prevention of hypertension development after EET-A and AAA treatment, some HR differences between groups will be observed. The strong association between the changes in both peripheral and central pressures and HR is a well-known phenomenon. However, this relationship is altogether very complex (Dalal et al., 2019).

A decrease in HR as a causative factor preventing an increase in blood pressure in young SHR treated with combined therapy is an attractive hypothesis but difficult to prove as there are almost no data indicating the role of EET and/or 20-HETE in heart rate regulation. To fill this gap, we used HRV analysis that could explain the role of cardiac autonomic activity in the hypotensive effect of EET-A + AAA in young rats. HRV, a variation in the time interval between heartbeats, is considered a reliable indicator of the interplay between the sympathetic and parasympathetic autonomic nervous systems (Rajendra Acharya et al., 2006; Sacha, 2014a).

Some observed changes in HRV could be simply related to the growth of the animals. The heart rate in young rats was reduced during the experiment (Figure 3B), which was not observed in adult rats (Figure 1B). This decrease was present in all groups of young animals, irrespective of the treatment, which could be interpreted as an effect of the maturation of the animals. In humans, the reduction of HR with age is a common developmental phenomenon, which, in turn, may influence HRV. The magnitude of HRV alterations during growth and development remains to be determined, as the majority of HRV studies are focused on changes in adult populations (Billman et al., 2019). In our experiments, we observed a clear elevation of NN interval, SDNN, VLF, and TP in young rats *vs*. the values obtained on the first day of the experiment, independently of treatment. Similar changes are absent in the adult groups. Moreover, it is suggested that the renin–angiotensin system is suppressed in SHR as a compensatory reaction against blood pressure elevation (Shiono and Sokabe, 1976). Virtanen *et al.* reported consistent inverse relationships between plasma renin activity and SDNN in hypertensive patients (Virtanen et al., 2003). Since we observed a comparable SDNN increase in all young groups (Supplementary Table S6), despite no SBP elevation in the EET-A + AAA group (Figure 3A), it seems that the hypotensive activity of the combined treatment is not related to changes in RAAS activity in young rats. However, in our previous study (Gawrys et al., 2020a), the combined treatment with EET-A and AAA lowered the renal levels of ANG II in adult rats, which suggests that suppression of RAAS could be one of the potential mechanisms of action of the combined treatment. In the present study, we have not measured the ANG II levels, which is a limitation of our study, and this should be addressed in future research.

Moreover, even similar changes in HR can provoke profoundly different values for HRV (Sacha and Pluta, 2005; Billman, 2013; Sacha, 2014b). Despite the same direction of the VLF band changes in all groups of young rats, the EET-A + AAA group exhibited a lower VLF value than the other groups at the end of the observation (day 28; Supplementary Table S7). So far, no practical application has been defined for VLF (Michel-Chávez et al., 2015), and this region of frequency band has been largely ignored (Shaffer et al., 2014). This component is regarded to reflect various activities associated with thermoregulatory, endocrine, heart, and vasomotor responses that may be mediated mostly by the sympathetic, but also potentially by the parasympathetic, nervous system (Akselrod et al., 1981; Claydon and Krassioukov, 2008; Dobrek et al., 2013). The guidelines from the Task Force of the European Society of Cardiology and the North American Society of Pacing and Electrophysiology (1996) suggest that VLF is a dubious measure that should be avoided. However, it has also been suggested that the VLF rhythm is intrinsically generated by the heart and that the amplitude and frequency of these oscillations are modulated by efferent sympathetic activity (Shaffer et al., 2014). Therefore, some studies have actually utilized VLF power as a reflection of sympathetic tone (Bhimani et al., 2011; D’Ascenzi et al., 2014; Brar et al., 2015) despite the fact that VLF as an indicator of sympathetic activity has not been conclusively confirmed (Thomas et al., 2019). Thus, the lower VLF in the EET-A + AAA group, in comparison to the control group, could be interpreted as a lower cardiac sympathetic activity and related to maintaining blood pressure within the normotensive range. However, this hypothesis is not supported by changes in normalized LF (Supplementary Table S7), which is a more widely accepted indicator of cardiac sympathetic activity (Ernst, 2017).

The analysis of the biochemical profile and the renal biomarkers suggest that, for the young rats, the treatment with EET-A and AAA was the most beneficial. The combined treatment significantly increased the level of urinary nitrate/nitrite, which are nitric oxide (NO) metabolites, and their excretion is commonly used as a marker of NO synthesis (Tsikas, 2005). It is well known for more than 2 decades that there is a strong correlation between hypertension and decreased NO production/bioavailability (Klahr, 2001; Hermann et al., 2006). This finding is in agreement with our previous study in adult SHR, in which we also reported an elevation of NO synthesis after the combined treatment (EET-A and AAA), which seemed to work in a synergistic manner since none of the substances given alone caused similar effects (Gawrys et al., 2020a). It was previously demonstrated that both AA metabolites (EETs and 20-HETE) are involved in the maintenance of endothelium and are responsible for its dysfunction. 20-HETE was shown to impair endothelial function through uncoupling of endothelial nitric oxide synthase (eNOS) *via* the activation of tyrosine kinase, a mitogen-activated protein kinase, and IκB kinase (Alonso-Galicia et al., 1997; Rocic and Schwartzman, 2018). On the other hand, EETs were shown to substantially increase eNOS expression and activity (Jiang et al., 2007), leading to an increased production of NO, which, in turn, might also inhibit *per se* the synthesis of 20-HETE (Alonso-Galicia et al., 1997). Considering our previous and current findings, it seems that the synergistic impact of both AAA and EET-A on NO production and bioavailability is one of the key mechanisms responsible for the antihypertensive effectiveness of the combined treatment. Moreover, these findings strongly support the hypothesis that NO bioavailability deficiency is critical for this type of multifactorial genetic form of hypertension observed in SHR, and the condition can be alleviated by the combined treatment of EET-A and AAA.

Another interesting observation is with regard the plasma level of IL-10, which is a multi-functional cytokine with a robust anti-inflammatory activity (Tinsley et al., 2010; Lima et al., 2016). It is common knowledge that inflammation is one of the key components in the pathophysiology of essential hypertension (Tanase et al., 2019). We observed a decreasing trend in both treated groups, which received EET-A alone and combined treatment with EET-A and AAA, possibly indicating that the state of inflammation was starting to diminish in those groups, possibly as an effect dependent on EET-A. The anti-inflammatory role of EETs has recently been thoroughly discussed by Imig (2019). On the other hand, none of the measured inflammatory parameters in the kidney changed during the treatment, which might suggest an improvement in the circulatory system rather than in the kidney.

We also assessed the daily UAE and creatinine clearance, well-established renal biomarkers which are still in common use in experimental as well as clinical practice (Griffin et al., 2020). We did not observe any changes between groups, and all the values were still in the normal range for healthy animals (Garrett et al., 2006). However, this is not very surprising for us. Many studies, including our own, indicate that especially UAE cannot be used as an early marker of kidney damage in SHR. We have previously shown that, in adult SHR with clearly visible severe morphological changes in the renal medulla, the levels of albumin in urine were still within the normal range, thus not indicating any renal damage (Gawrys et al., 2018).

Moreover, we also measured the excretion rate of VEGF-A, which calls for special attention. The analysis revealed that VEGF-A excretion decreased after treatment with EET-A + AAA in young rats, which surprisingly contradicts our previous findings in adult SHR (Gawrys et al., 2020a). In our original study, we observed an increase of this biomarker (after treatment with EET-A and AAA in adult SHR), which we have considered as a beneficial sign of improved kidney function, especially considering that it was shown that VEGF-A stimulates the synthesis of NO (Robinson et al., 2010). Our current results are somehow contradictory to our original hypothesis. However, to date, the precise role of VEGF-A in blood pressure regulation remains not fully understood, and there are many contradictory reports regarding its mechanisms of action and pathways—for instance, it was shown that the overexpression of VEGF (in particular, the 164 isoform) in podocytes resulted in collapsing glomerulopathy in mice (Eremina et al., 2003; Schrijvers et al., 2004). The VEGF blockage also proved to be favorable for diabetes-associated renal changes, which suggest a rather detrimental role of this growth factor in diabetic nephropathy (Schrijvers et al., 2004). Additionally, it suggests that the role of VEGF-A in normal physiological conditions in healthy animals is limited (Schrijvers et al., 2004). Therefore, we are not able to unequivocally draw any firm conclusions; we can merely suggest that the role of VEGF-A in hypertension development in young SHR is not as crucial as previously thought.

### Summary, Conclusions, and Limitations

We confirmed our previous finding that the antihypertensive treatment unaccompanied by the 14,15-EET analog (EET-A) is not effective in adult SHR, even at a substantially higher dosage, as well as in young rats. We hypothesize that the hypertensive activity of EET-A in the brain might counterbalance the peripheral effects of the analog. To test this hypothesis, more extensive studies on BBB permeability in SHR are necessary.

However, the combined treatment with EET-A and AAA proved to be very beneficial for young SHR, which remained normotensive during the 4 weeks of drug administration. In the future, it would be beneficial to assess the effectiveness of AAA administered alone in young rats and to verify our previous hypothesis about the synergistic activity of both substances observed in adult rats. Despite our efforts to elucidate in more detail the actual mechanism of the combined treatment of EET-A and AAA, it seems that it is not directly related to RAAS and SNS activity, and more studies are still needed. It seems that the antihypertensive activity of EET-A + AAA is linked to the increased bioavailability of nitric oxide, which is consistent with our previous findings.

## Data Availability

The raw data supporting the conclusions of this article will be made available by the authors without undue reservation.
