# Supplementary material for: Epoxyeicosatrienoic Acid Analog and 20-HETE Antagonist Combination Prevent Hypertension Development in Spontaneously Hypertensive Rats
Source: Front Pharmacol. 2022 Jan 17;12:798642. doi: 10.3389/fphar.2021.798642 (PMC8802114; doi:10.3389/fphar.2021.798642)
Supplement: Supplementary file 1 [file DataSheet1.docx]

Supplementary Material


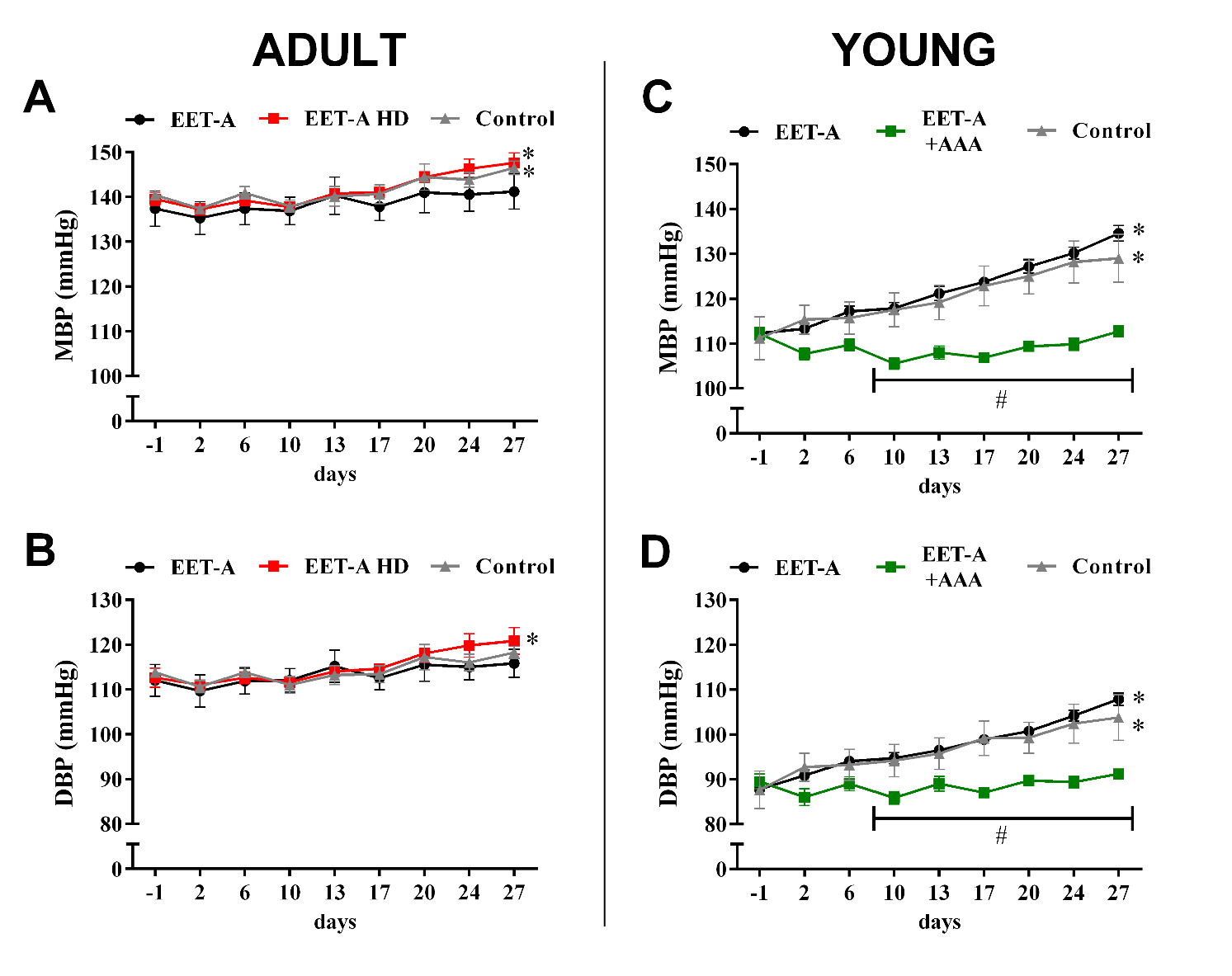


**Supplementary Figure S1**

Time course of **A, C:** mean and **B, D:** diastolic blood pressure (MBP, DBP respectively) in **ADULT** (**A,B**) and **YOUNG** (**C,D**) spontaneously hypertensive rats (SHR) receiving epoxyeicosatrienoic acid analog in two doses: 10 mg/kg/day (EET-A) and 40 mg/kg/day (EET-A HD), or combined with 20-HETE receptor antagonist (EET-A+AAA) both at a dose of 10 mg/kg/day; control groups received water (n=6-7 in each group). Values are expressed as means ± SEM. *p<0.05 vs baseline values (day -1) within each group by two-way analysis of variance followed by Bonferroni's multiple comparisons test; # p<0.05 EET-A+AAA vs EET-A and control group in the same time point by two-way analysis of variance followed by Tukey post hoc test.

**Supplementary Table S1**

Time-domain HRV variables calculated from data collected on day 0 and 28^th^ in **ADULT** spontaneously hypertensive rats (SHR) receiving oral treatment (in drinking water) with epoxyeicosatrienoic acid analog in a dose of 10 mg/kg/day (EET-A) or 40 mg/kg/day (EET-A HD).

| **Parameter** |  | **Day** | |  |
| --- | --- | --- | --- | --- |
|  | **Group** | **0** | **28** | Δ |
| **NN**  **(ms)** | *EET-A* | 184.26±2.21 | 186.72±2.77 | 2.46±4.03 |
|  | *EET-A HD* | 184.81±3.45 | 187.22±3.53 | 2.41±2.88 |
|  | *Control* | 180.62±1.66 | 189.63±3.09 | 9.01±3.19 |
| **SDNN**  **(ms)** | *EET-A* | 20.86±0.34 | 22.31±0.36 | 1.45±0.48 |
|  | *EET-A HD* | 22.81±0.81 | 23.93±0.71 | 1.12±0.81 |
|  | *Control* | 21.82±0.76 | 24.27±0.41 | 2.45±0.81 |
| **RMSSD**  **(ms)** | *EET-A* | 4.69±0.36 | 5.60±0.51 | 0.90±0.27 |
|  | *EET-A HD* | 5.51±0.70 | 5.50±0.56 | -0.01±0.31 |
|  | *Control* | 5.31±0.46 | 5.46±0.41 | 0.15±0.41 |
| **pNN5**  **(%)** | *EET-A* | 17.61±2.26 | 23.11±3.22 | 5.50±2.42 |
|  | *EET-A HD* | 24.10±6.05 | 25.21±4.66 | 1.11±2.21 |
|  | *Control* | 21.62±4.98 | 25.46±3.30 | 3.84±2.37 |

*Normal beat-to-beat intervals (NN); standard deviation of NN intervals (SDNN);square root of the mean square successive differences between successive normal intervals (RMSSD);percentage of normal consecutive NN intervals differing by more than 5 ms (pNN5); values are expressed as means ± SEM (n = 5 in each group).*

**Supplementary Table S2**

Frequency-domain HRV variables calculated from data collected on day 0 and 28^th^ in **ADULT** spontaneously hypertensive rats (SHR) receiving oral treatment (in drinking water) with epoxyeicosatrienoic acid analog in a dose of 10 mg/kg/day (EET-A) or 40 mg/kg/day (EET-A HD)

| **Parameter** |  | **Day** | |  |
| --- | --- | --- | --- | --- |
|  | **Group** | **0** | **28** | Δ |
| **VLF**  **(Hz)** | *EET-A* | 25.62±1.61 | 30.91±3.79 | 5.29±3.78 |
|  | *EET-A HD* | 34.39±3.60 | 44.05±2.45 # † | 9.66±2.35 |
|  | *Control* | 24.28±2.35 | 32.03±2.70 | 7.75±1.95 |
| **Normalized LF**  **(nu)** | *EET-A* | 0.34±0.03 | 0.30±0.03 | -0.04±0.02 |
|  | *EET-A HD* | 0.33±0.06 | 0.37±0.03 | 0.04±0.03 |
|  | *Control* | 0.27±0.05 | 0.27±0.01 | 0.00±0.04 |
| **Normalized HF**  **(nu)** | *EET-A* | 0.66±0.03 | 0.70±0.03 | 0.04±0.02 |
|  | *EET-A HD* | 0.67±0.06 | 0.63±0.03 | -0.04±0.03 |
|  | *Control* | 0.73±0.05 | 0.73±0.01 | 0.00±0.04 |
| **LF-HF ratio**  **(%)** | *EET-A* | 0.58±0.07 | 0.49±0.06 | -0.09±0.04 |
|  | *EET-A HD* | 0.61±0.15 | 0.65±0.08 | 0.04±0.07 |
|  | *Control* | 0.44±0.11 | 0.44±0.04 | 0.00±0.10 |
| **TP**  **(ms^2^)** | *EET-A* | 29.49±0.95 | 35.34±3.95 | 5.85±4.01 |
|  | *EET-A HD* | 40.29±4.24 # † | 49.58±3.37 # † | 9.29±2.32 |
|  | *Control* | 26.73±1.92 | 35.47±2.04 | 8.74±2.03 |

*VLF – very low frequency band (under 0.2 Hz); normalized LF – low frequency band (0.2-0.74 Hz) expressed as the relative value of each power component in proportion to the total power minus the very low-frequency; normalized HF – high frequency band (0.74-2.5) expressed as the relative value of each power component in proportion to the total power minus the very low-frequency; TP – total spectral power; nu – normalized units. Values are expressed as means ± SEM (n=5 in each group);
# vs EET-A on the same day, † vs control group on the same day, one way ANOVA with Tukey post hoc test.*

**Supplementary Table S3**

Metabolic parameters measured on 0, 7, 14, 21 and 28th day in **ADULT** spontaneously hypertensive rats (SHR) receiving oral treatment (in drinking water) with epoxyeicosatrienoic acid analog in a dose of 10 mg/kg/day (EET-A) or 40 mg/kg/day (EET-A HD)

| **Parameter** |  | **Day** | | | | |
| --- | --- | --- | --- | --- | --- | --- |
|  | **Group** | **0** | **7** | **14** | **21** | **28** |
| **Body weight**  **(g)** | *EET-A* | 320±10 | 328±8 | 338±8 | 345±8 | 356±7 |
|  | *EET-A HD* | 309±11 | 323±10 | 334±9 | 341±8 | 346±8 |
|  | *Control* | 311±7 | 325±7 | 331±8 | 341±6 | 344±6 |
| **Diuresis**  **(ml/24h)** | *EET-A* | 18±2 | 20±2 | 21±4 | 19±2 | 21±3 |
|  | *EET-A HD* | 19±2 | 16±1 | 17±1 | 20±1 | 21±2 |
|  | *Control* | 15±1 | 22±3 | 24±3 | 32±6 | 23±2 |
| **Water inatake**  **(ml/24h)** | *EET-A* | 34±4 | 39±4 | 42±3 | 40±3 | 44±3 |
|  | *EET-A HD* | 35±2 | 33±2 | 37±2 | 40±2 | 36±4 |
|  | *Control* | 27±5 | 33±3 | 40±3 | 41±3 | 42±3 |
| **Food intake**  **(g/24h)** | *EET-A* | 16±3 | 18±1 | 21±1 | 21±1 | 22±1 |
|  | *EET-A HD* | 19±2 | 21±1 | 23±1 | 23±1 | 20±2 |
|  | *Control* | 13±4 | 14±3 | 19±1 | 19±2 | 22±1 |
| **Feaces**  **(g/24h)** | *EET-A* | 12±1 | 12±1 | 14±1 | 12±1 | 14±2 |
|  | *EET-A HD* | 11±1 | 14±1 | 13± | 12±1 | 12±1 |
|  | *Control* | 9±2 | 10±1 | 10±1 | 10±2 | 12±1 |

*Values are expressed as means ± SEM (n=6 in each group)*

**Supplementary Table S4**

Plasma and excretory parameters measured on 0, 7, 14, 21 and 28th day in **ADULT** spontaneously hypertensive rats (SHR) receiving oral treatment (in drinking water) with epoxyeicosatrienoic acid analog in a dose of 10 mg/kg/day (EET-A) or 40 mg/kg/day (EET-A HD)

| **Parameter** |  | **Day** | | | | |
| --- | --- | --- | --- | --- | --- | --- |
|  | **Group** | **0** | **7** | **14** | **21** | **28** |
| **Haematocrit (%)** | *EET-A* | 48±1 | 47±1 | 47±1 | 49±0 | 49±1 |
|  | *EET-A HD* | 49±0 | 48±0 | 48±0 | 50±0 | 50±0 |
|  | *Control* | 49±1 | 49±1 | 49±0 | 49±0 | 49±0 |
| **Plasma osmolality**  **(mOsm/l)** | *EET-A* | 303±1 | 310±1 | 309±1 | 304±3 | 305±3 |
|  | *EET-A HD* | 309±3 | 306±1 | 308±2 | 305±1 | 305±1 |
|  | *Control* | 313±4 | 311±3 | 308±1 | 312±3 | 307±2 |
| **Plasma sodium**  **(mmol/l)** | *EET-A* | 135±2 | 130±2 | 133±1 | 133±3 | 133±2 |
|  | *EET-A HD* | 132±1 | 132±1 | 130±2 | 131±2 | 131±1 |
|  | *Control* | 131±1 | 128±1 | 130±3 | 129±2 | 132±2 |
| **Total solute excretion**  **(µOsm/min)** | *EET-A* | 11.2±1.3 | 12.6±0.6 | 14.3±2.9 | 11.4±1.7 | 11.9±2.0 |
|  | *EET-A HD* | 17.3±2.5 | 12.8±0.7 | 12.8±1.6 | 11.0±0.6 | 16.9±1.9 |
|  | *Control* | 14.8±1.7 | 17.8±2.3 | 14.9±1.0 | 18.4±2.4 | 16.6±1.6 |
| **Sodium excretion**  **(µmol/min)** | *EET-A* | 0.6±0.1 | 0.5±0.1 | 0.5±0.1 | 0.5±0.2 | 0.3±0.1 |
|  | *EET-A HD* | 0.7±0.2 | 0.5±0.1 | 0.6±0.1 | 0.4±0.1 | 0.6±0.2 |
|  | *Control* | 0.6±0.1 | 0.6±0.2 | 0.7±0.1 | 0.6±0.2 | 0.8±0.1 |

*Values are expressed as means ± SEM (n=6 in each group)*

**Supplementary Table S5**

Organ weights collected in the end of the four week observation from **ADULT** spontaneously hypertensive rats (SHR) receiving oral treatment (in drinking water) with epoxyeicosatrienoic acid analog in a dose of 10 mg/kg/day (EET-A) or 40 mg/kg/day (EET-A HD)

|  | | **HW (g)** | **LV (g)** | **RK (g)** | **LK (g)** | **HW/BW (mg/g)** | **LV/BW (mg/g)** | **LV/HW (g/g)** |  |  |
| --- | --- | --- | --- | --- | --- | --- | --- | --- | --- | --- |
| **Control** | 1.35±0.04 | | 0.99±0.02 | 1.16±0.02 | 1.17±0.03 | 3.94±0.06 | 2.90±0.04 | 0.74±0.01 |  |  |
| **EET-A** | 1.43±0.07 | | 0.98±0.04 | 1.25±0.05 | 1.29±0.04 | 4.04±0.20 | 2.76±0.09 | 0.69±0.03 |  |  |
| **EET-A HD** | 1.28±0.05 | | 0.99±0.03 | 1.12±0.03 | 1.14±0.03 | 3.71±0.09 | 2.85±0.04 | 0.77±0.01 |  |  |

*Heart weight (HW), left ventricle (LV) weight, right kidney weight (RK), left kidney weight (LK), body weight (BW); Values are expressed as means ± SEM (n = 6 in each group); NS*

**Supplementary Table S6**

Concentration of 23 cytokines in the kidney of **ADULT** spontaneously hypertensive rats (SHR) receiving oral treatment (in drinking water) with epoxyeicosatrienoic acid analog in a dose of 40 mg/kg/day (EET-A HD) and control.

| **Parameter**  **(mg/g)** | **EET-A HD** | **CONTROL** |
| --- | --- | --- |
| **IL-1α** | 3.87±0.19 | 3.02±0.36 |
| **IL-1β** | 3.02±0.21 | 2.43±0.23 |
| **IL-2** | 97.47±5.40***** | 73.83±7.44 |
| **IL-4** | 3.05±0.14***** | 2.38±0.25 |
| **IL-5** | 3.63±0.21 | 2.84±0.32 |
| **IL-6** | 12.48±0.67 | 10.08±1.09 |
| **IL-7** | 3.77±0.31 | 3.10±0.28 |
| **IL-10** | 4.89±0.38 | 4.03±0.40 |
| **IL-12 (p70)** | 9.07±0.49 | 7.10±0.76 |
| **IL-13** | 2.75±0.16 | 2.15±0.23 |
| **IL-17A** | 0.52±0.03***** | 0.40±0.04 |
| **IL-18** | 55.95±4.30 | 45.49±4.53 |
| **G-CSF** | 0.08±0.01***** | 0.06±0.01 |
| **GM-CSF** | 3.01±0.23 | 2.48±0.22 |
| **GRO/K** | 1.31±0.06 | 1.01±0.11 |
| **IFN-γ** | 4.21±0.19***** | 3.19±0.33 |
| **M-CSF** | 0.32±0.02***** | 0.25±0.03 |
| **MCP-1** | 10.72±0.83 | 8,76±0.84 |
| **MIP-1α** | 0.40±0.02 | 0.32±0.03 |
| **MIP-3α** | 0.32±0.02***** | 0.23±0.02 |
| **RANTES** | 1.64±0.14 | 1.28±0.13 |
| **TNF- α** | 21.31±1.16 | 17.22±1.76 |
| **VEGF** | 4.97±0.26 | 3.93±0.40 |

*Interleukin-1α (IL-1α), interleukin-1β (IL-1β), interleukin-2 (IL-2), interleukin-4 (IL-4), interleukin-5 (IL-5), interleukin-6 (IL-6), interleukin-7 (IL-7), interleukin-10 (IL-10), interleukin-12 (IL-12), interleukin-13 (IL-13), interleukin-17A (IL-17A), interleukin-18 (IL-18),* *granulocyte colony-stimulating factor (G-CSF),* *granulocyte-macrophage colony-stimulating factor (GM-CSF),* *keratinocyte chemoattractant (KC)/ growth-regulated oncogene (GRO) chemokines (GRO/K),* *interferon gamma (IFN-γ****),*** *macrophage colony stimulating factor (M-CSF), monocyte chemoattractant protein-1 (MCP-1), macrophage inflammatory protein 1α (MIP-1α), macrophage inflammatory protein 3α (MIP-3α), regulated on activation, normal T-cell expressed and secreted (RANTES), tumor necrosis factor- α (TNF- α), vascular endothelial growth factor (VEGF). Values are expressed as means ± SEM (n = 5 in each group).******** *p<0.05 vs control group, unpaired t-test.*

**Supplementary Table S7**

Time-domain HRV variables calculated from data collected on day 0 and 28^th^ in **YOUNG** spontaneously hypertensive rats (SHR) receiving oral treatment (in drinking water) with epoxyeicosatrienoic acid analog (EET-A) alone or combined with AAA, a 20-hydroxyeicosatetraenoic receptor antagonist (EET-A+AAA) both in a dose of 10 mg/kg/day

| **Parameter** |  | **Day** | |  |
| --- | --- | --- | --- | --- |
|  | **Group** | **0** | **28** | Δ |
| **NN**  **(ms)** | *EET-A* | 139.61±1.88 | 176.66±2.43 * | 37.05±2.42 |
|  | *EET-A+AAA* | 136.29±1.35 | 165.30±1.43 * # | 29.01±2.26 |
|  | *Control* | 138.04±2.26 | 170.06±0.45 * | 32.02±1.88 |
| **SDNN**  **(ms)** | *EET-A* | 13.23±0.57 | 19.84±0.41 * | 6.61±0.41 |
|  | *EET-A+AAA* | 13.82±0.34 | 18.61±0.63 * | 4.79±0.67 |
|  | *Control* | 13.64±0.59 | 19.73±0.90 * | 6.09±1.16 |
| **RMSSD**  **(ms)** | *EET-A* | 4.45±0.34 | 6.54±0.57 * | 2.09±0.71 |
|  | *EET-A+AAA* | 3.58±0.40 | 5.57±0.62 | 1.99±0.23 |
|  | *Control* | 3.89±0.59 | 5.07±0.56 | 1.18±0.48 |
| **pNN5**  **(%)** | *EET-A* | 3.53±0.93 | 19.38±3.83 * | 15.85±3.40 |
|  | *EET-A+AAA* | 4.90±2.13 | 17.36±4.00 | 12.46±2.00 |
|  | *Control* | 2.08±0.38 | 9.51±1.44 | 7.43±1.42 |

*Normal beat-to-beat intervals (NN); standard deviation of NN intervals (SDNN);square root of the mean square successive differences between successive normal intervals (RMSSD); percentage of normal consecutive NN intervals differing by more than 5 ms (pNN5). Values are expressed as means ± SEM (n = 6 in each group); * vs day 0, # vs EET-A on the same day, one-way ANOVA with Tukey post hoc test.*

**Supplementary Table S8**

Frequency-domain HRV variables calculated from data collected on day 0 and 28^th^ in **YOUNG** spontaneously hypertensive rats (SHR) receiving oral treatment (in drinking water) with epoxyeicosatrienoic acid analog (EET-A) alone or combined with AAA, a 20-hydroxyeicosatetraenoic receptor antagonist (EET-A+AAA) both in a dose of 10 mg/kg/day

| **Parameter** |  | **Day** | |  | |
| --- | --- | --- | --- | --- | --- |
|  | **Group** | **0** | **28** | | Δ |
| **VLF**  **(Hz)** | *EET-A* | 10.57±0.18 | 25.25±1.41 * | | 14.68±1.39 |
|  | *EET-A+AAA* | 10.98±0.62 | 20.29±2.02 * † | | 9.31±2.35 |
|  | *Control* | 9.93±0.99 | 26.83±1.61 * | | 16.90±2.32 |
| **Normalized LF**  **(nu)** | *EET-A* | 0.30±0.03 | 0.26±0.01 | | -0.04±0.02 |
|  | *EET-A+AAA* | 0.35±0.02 | 0.32±0.05 | | -0.03±0.04 |
|  | *Control* | 0.41±0.01 | 0.34±0.02 | | -0.07±0.03 |
| **Normalized HF**  **(nu)** | *EET-A* | 0.70±0.03 | 0.74±0.01 | | 0.04±0.02 |
|  | *EET-A+AAA* | 0.65±0.02 | 0.68±0.05 | | 0.03±0.04 |
|  | *Control* | 0.60±0.01 | 0.66±0.02 | | 0.06±0.03 |
| **LF-HF ratio**  **(%)** | *EET-A* | 0.47±0.06 | 0.37±0.02 | | -0.10±0.04 |
|  | *EET-A+AAA* | 0.61±0.04 | 0.54±0.11 | | -0.07±0.10 |
|  | *Control* | 0.73±0.05 | 0.56±0.05 | | -0.17±0.07 |
| **TP**  **(ms^2^)** | *EET-A* | 12.34±0.85 | 29.46±1.97 * | | 17.12±2.23 |
|  | *EET-A+AAA* | 12.71±0.85 | 24.82±1.88 * | | 12.11±2.30 |
|  | *Control* | 12.50±1.42 | 26.27±1.76 * | | 13.77±2.65 |

*VLF – very low frequency band (under 0.2 Hz); normalized LF – low frequency band (0.2-0.74 Hz) expressed as the relative value of each power component in proportion to the total power minus the very low-frequency; normalized HF – high frequency band (0.74-2.5) expressed as the relative value of each power component in proportion to the total power minus the very low-frequency; TP – total spectral power; nu – normalized units. Values are expressed as means ± SEM (n = 6 in each group). * vs day 0, † vs control group on the same day, one-way ANOVA with Tukey post hoc test.*

**Supplementary Table S9**

Metabolic parameters measured on 0, 7, 14, 21 and 28th day in **YOUNG** spontaneously hypertensive rats (SHR) receiving oral treatment (in drinking water) with epoxyeicosatrienoic acid analog (EET-A) alone or combined with AAA, a 20-hydroxyeicosatetraenoic receptor antagonist (EET-A+AAA) both in a dose of 10 mg/kg/day

| **Parameter** |  | **Day** | | | | |
| --- | --- | --- | --- | --- | --- | --- |
|  | **Group** | **0** | **7** | **14** | **21** | **28** |
| **Body weight**  **(g)** | *EET-A* | 138±7 | 178±7 | 212±7 | 238±6 | 257±6 |
|  | *EET-A+AAA* | 113±3 | 148±4 | 186±4 | 212±5 | 234±5 |
|  | *Control* | 117±5 | 150±7 | 185±6 | 214±6 | 237±5 |
| **Diuresis**  **(ml/24h)** | *EET-A* | 9±1 | 11±1 | 17±1 | 19±2 | 18±3 |
|  | *EET-A+AAA* | 5±1 | 9±1 | 14±1 | 15±1 | 14±1 |
|  | *Control* | 10±1 | 14±1 | 18±1 | 18±1 | 22±2 |
| **Water intake**  **(ml/24h)** | *EET-A* | 24±2 | 31±2 | 34±2 | 38±3 | 37±4 |
|  | *EET-A+AAA* | 23±1 | 26±1 | 33±2 | 31±3 | 36±2 |
|  | *Control* | 27±1 | 30±1 | 34±1 | 36±1 | 41±2 |
| **Food intake**  **(g/24h)** | *EET-A* | 18±1 | 21±1 | 21±1 | 22±1 | 22±1 |
|  | *EET-A+AAA* | 17±1 | 20±1 | 22±1 | 24±1 | 25±1 |
|  | *Control* | 15±1 | 17±1 | 20±1 | 21±0 | 21±1 |
| **Feaces**  **(g/24h)** | *EET-A* | 9±1 | 10±1 | 12±1 | 11±1 | 12±1 |
|  | *EET-A+AAA* | 8±0 | 9±1 | 11±1 | 12±1 | 13±1 |
|  | *Control* | 8±0 | 9±0 | 11±1 | 10±1 | 10±1 |

*Values are expressed as means ± SEM (n = 6 in each group)*

**Supplementary Table S10**

Plasma and excretory parameters measured on 0, 7, 14, 21 and 28th in **YOUNG** spontaneously hypertensive rats (SHR) receiving oral treatment (in drinking water) with epoxyeicosatrienoic acid analog (EET-A) alone or combined with AAA, a 20-hydroxyeicosatetraenoic receptor antagonist (EET-A+AAA) both in a dose of 10 mg/kg/day

| **Parameter** |  | **Day** | | | | |
| --- | --- | --- | --- | --- | --- | --- |
|  | **Group** | **0** | **7** | **14** | **21** | **28** |
| **Haematocrit (%)** | *EET-A* | 42±0 | 45±1 | 46±1 | 49±1 | 49±0 |
|  | *EET-A+AAA* | 44±1 | 44±1 | 43±1 | 47±0 | 47±1 |
|  | *Control* | 43±0 | 44±1 | 46±0 | 47±0 | 49±1 |
| **Plasma osmolality**  **(mOsm/l)** | *EET-A* | 302±1 | 298±1 | 303±3 | 293±3 | 302±1 |
|  | *EET-A+AAA* | 300±3 | 297±1 | 297±1 | 288±1 | 293±1 |
|  | *Control* | 300±1 | 295±2 | 303±2 | 295±3 | 302±2 |
| **Plasma sodium**  **(mmol/l)** | *EET-A* | 131±1 | 133±1 | 129±1 | 131±1 | 132±2 |
|  | *EET-A+AAA* | 132±0 | 131±1 | 135±0 | 132±1 | 134±1 |
|  | *Control* | 128±1 | 131±1 | 132±1 | 131±1 | 131±2 |
| **Total solute excretion**  **(µOsm/min)** | *EET-A* | 6.2±0.6 | 8.9±1.2 | 15.4±1.5 | 15.7±2.8 | 14.5±3.5 |
|  | *EET-A+AAA* | 6.0±0.8 | 9.1±2.3 | 10.7±0.4 | 10.0±1.1 | 12.3±1.8 |
|  | *Control* | 6.0±0.6 | 8.7±1.4 | 10.9±1.4 | 14.0±1.7 | 14.7±1.4 |
| **Sodium excretion**  **(µmol/min)** | *EET-A* | 0.5±0.1 | 0.7±0.1 | 0.9±0.1 | 1.0±0.2 | 0.7±0.2 |
|  | *EET-A+AAA* | 0.6±0.1 | 0.9±0.1 | 0.9±0.1 | 1.0±0.1 | 0.9±0.2 |
|  | *Control* | 0.7±0.1 | 1.0±0.2 | 1.1±0.1 | 1.0±0.1 | 1.1±0.2 |

*Values are expressed as means ± SEM (n = 6 in each group)*

**Supplementary Table S11**

Organ weights collected in the end of the four week observation in **YOUNG** spontaneously hypertensive rats (SHR) receiving oral treatment (in drinking water) with epoxyeicosatrienoic acid analog (EET-A) alone or combined with AAA, a 20-hydroxyeicosatetraenoic receptor antagonist (EET-A+AAA) both in a dose of 10 mg/kg/day

|  | **HW (g)** | **LV (g)** | **RK (g)** | **LK (g0** | **HW/BW (mg/g)** | **LV/BW (mg/g)** | **LV/HW (g/g)** |  |  |
| --- | --- | --- | --- | --- | --- | --- | --- | --- | --- |
| **Control** | 1.06±0.04 | 0.77±0.03 | 0.97±0.03 | 0.98±0.02 | 4.10±0.09 | 2.95±0.06 | 0.72±0.01 |  |  |
| **EET-A** | 0.97±0.02 | 0.73±0.04 | 1.01±0.03 | 1.02±0.04 | 4.03±0.14 | 3.02±0.16 | 0.75±0.02 |  |  |
| **EET-A+AAA** | 0.90±0.04 | 0.64±0.03 | 0.99±0.03 | 0.97±0.04 | 3.87±0.15 | 2.75±0.13 | 0.71±0.01 |  |  |

*Heart weight (HW), left ventricle weight (LV), right kidney weight (RK), left kidney weight (LK), body weight (BW); values are expressed as means ± SEM (n = 6 in each group); NS*

**Supplementary Table S12**

Concentration of 23 cytokines in the kidney of **YOUNG** spontaneously hypertensive rats (SHR) receiving oral treatment (in drinking water) with epoxyeicosatrienoic acid analog (EET-A) combined with AAA, a 20-hydroxyeicosatetraenoic receptor antagonist (EET-A+AAA) in a dose of 10 mg/kg/day and control.

| **Parameter**  **(mg/g)** | **EET-A + AAA** | **CONTROL** |
| --- | --- | --- |
| **IL-1α** | 3.81±0.26 | 3.01±0.17 |
| **IL-1β** | 2.81±0.15 | 3.05±0.18 |
| **IL-2** | 89.62±8.18 | 91.51±6.84 |
| **IL-4** | 2.61±0.21 | 2.60±0.14 |
| **IL-5** | 3.15±0.19 | 3.15±0.13 |
| **IL-6** | 11.09±0.75 | 11.21±0.59 |
| **IL-7** | 3.65±0.23 | 3.90±0.23 |
| **IL-10** | 4.71±0.27 | 5.04±0.29 |
| **IL-12 (p70)** | 7.81±0.60 | 7.75±0.47 |
| **IL-13** | 2.42±0.17 | 2.35±0.16 |
| **IL-17A** | 0.44±0.03 | 0.44±0.02 |
| **IL-18** | 52.34±3.48 | 56.42±3.49 |
| **G-CSF** | 0.06±0.01 | 0.06±0.01 |
| **GM-CSF** | 2.82±0.18 | 3.04±0.18 |
| **GRO/K** | 1.14±0.07 | 1.16±0.03 |
| **IFN-γ** | 3.65±0.24 | 3.87±0.16 |
| **M-CSF** | 0.26±0.02 | 0.25±0.01 |
| **MCP-1** | 10.15±0.74 | 10.53±0.70 |
| **MIP-1α** | 0.37±0.03 | 0.39±0.02 |
| **MIP-3α** | 0.29±0.02 | 0.31±0.03 |
| **RANTES** | 1.71±0.05 | 1.91±0.11 |
| **TNF- α** | 19.57±0.94 | 21.57±1.03 |
| **VEGF** | 4.22±0.30 | 4.34±0.23 |

*Interleukin-1α (IL-1α), interleukin-1β (IL-1β), interleukin-2 (IL-2), interleukin-4 (IL-4), interleukin-5 (IL-5), interleukin-6 (IL-6), interleukin-7 (IL-7), interleukin-10 (IL-10), interleukin-12 (IL-12), interleukin-13 (IL-13), interleukin-17A (IL-17A), interleukin-18 (IL-18),* *granulocyte colony-stimulating factor (G-CSF),* *granulocyte-macrophage colony-stimulating factor (GM-CSF),* *keratinocyte chemoattractant (KC)/ growth-regulated oncogene (GRO) chemokines (GRO/K),* *interferon gamma (IFN-γ****),*** *macrophage colony stimulating factor (M-CSF), monocyte chemoattractant protein-1 (MCP-1), macrophage inflammatory protein 1α (MIP-1α), macrophage inflammatory protein 3α (MIP-3α), regulated on activation, normal T-cell expressed and secreted (RANTES), tumor necrosis factor- α (TNF- α), vascular endothelial growth factor (VEGF).* *Values are expressed as means ± SEM* *(n = 5 in each group), NS*
